# Impaired Phagocytosis in Dendritic Cells From Pediatric Patients With Type 1 Diabetes Does Not Hamper Their Tolerogenic Potential

**DOI:** 10.3389/fimmu.2019.02811

**Published:** 2019-11-28

**Authors:** Silvia Rodriguez-Fernandez, Marta Murillo, Adrian Villalba, David Perna-Barrull, Mary Cano-Sarabia, Laia Gomez-Muñoz, Eva Aguilera, Daniel Maspoch, Federico Vazquez, Joan Bel, Marta Vives-Pi

**Affiliations:** ^1^Immunology Section, Germans Trias i Pujol Research Institute, Autonomous University of Barcelona, Badalona, Spain; ^2^Pediatrics Section, Germans Trias i Pujol University Hospital, Badalona, Spain; ^3^Catalan Institute of Nanoscience and Nanotechnology, CSIC and the Barcelona Institute of Science and Technology, Bellaterra, Spain; ^4^Endocrinology Section, Germans Trias i Pujol University Hospital, Badalona, Spain; ^5^Catalan Institution for Research and Advanced Studies (ICREA), Barcelona, Spain; ^6^Biomedical Research Center in Diabetes Network and Associated Metabolic Diseases (CIBERDEM), Carlos III Health Institute (ISCiii), Madrid, Spain

**Keywords:** dendritic cells, immune tolerance, type 1 diabetes, phagocytosis, autoimmunity

## Abstract

Type 1 diabetes (T1D) is prompted by defective immunological tolerance, an event in which dendritic cells (DCs) are crucial as immune response orchestrators. In fact, they contribute to maintaining tolerance to self-antigens, but they can also prompt an immunogenic response against them, leading to autoimmunity. Countless factors can potentially impact on the proper functionality of the DCs, which range from altered subset distribution, impaired phagocytic function to abnormal gene expression. Moreover, in T1D, metabolic dysregulation could impair DC functions as well. Indeed, since T1D clinical course is likely to be more aggressive in children and adolescents and entails severe dysglycemia, the aim of this study was to analyze circulating DCs subpopulations in pediatric T1D at different stages, as well as to characterize their phagocytosis ability and tolerance induction potential. Thus, pediatric patients newly diagnosed with T1D, with established disease and control subjects were recruited. Firstly, DCs subsets from peripheral blood were found quantitatively altered during the first year of disease, but recovered in the second year of progression. Secondly, to study the tolerogenic functionality of DCs, liposomes with phosphatidylserine (PS) were designed to mimic apoptotic beta cells, which are able to induce tolerance, as previously demonstrated by our group in DCs from adult patients with T1D. In this study, monocyte-derived DCs from pediatric patients with T1D and control subjects were assessed in terms of PS-liposomes capture kinetics, and transcriptional and phenotypic changes. DCs from pediatric patients with T1D were found to phagocyte PS-liposomes more slowly and less efficiently than DCs from control subjects, inversely correlating with disease evolution. Nonetheless, the transcription of PS receptors and immunoregulatory genes, cytokine profile, and membrane expression of immunological markers in DCs was consistent with tolerogenic potential after PS-liposomes phagocytosis. In conclusion, T1D progression in childhood entails altered peripheral blood DCs subsets, as well as impaired DCs phagocytosis, although tolerance induction could still function optimally. Therefore, this study provides useful data for patient follow-up and stratification in immunotherapy clinical trials.

## Introduction

Type 1 diabetes (T1D) is prompted by defective immunological tolerance to beta cells, resulting in their destruction by autoreactive lymphocytes. Although the triggering factors causing their activation remain elusive, antigen-presenting cells (APCs) are known to contribute to this activation, and so, to the development of the disease. Among APCs, dendritic cells (DCs), as orchestrators of the immune response, play a crucial role in the process. On the one side, they can induce and preserve self-tolerance but, on the other side, they can aid in its breach. Indeed, many factors could potentially impact on the proper functionality of the DCs, ranging from altered subset distribution, impaired phagocytic function, abnormal gene expression to defective functionality. As a case in point, many studies have linked the deficient removal of apoptotic beta cells performed by phagocytes to the initiation of autoimmunity (1). In fact, beta cells experience massive peaks of apoptosis, not only as a consequence of the autoimmune attack (2, 3), but also in physiological islet remodeling during the perinatal period (4, 5).

Plasma membrane integrity is maintained during apoptosis, thus preventing the release of immunogenic signals (6). This process, accompanied by fast apoptotic cell clearance by phagocytes—termed efferocytosis—, allows apoptosis to be immunomodulatory, which is essential in the maintenance of homeostasis. Apoptotic cell removal is mediated by highly conserved receptors and ligands, intracellular signaling, and engulfment mechanisms. One of the most well-characterized hallmarks of apoptosis is the exposure of phosphatidylserine (PS) on the membrane of apoptotic cells (7). PS interacts with several PS receptors —e.g., MerTK, CD36, and MFG-E8— on APCs, which promote efferocytosis, and this process confers biological advantages, such as peripheral self-tolerance induction (8, 9). Indeed, efferocytosis prompts immunomodulatory effects in phagocytes, which drive a tolerogenic antigen presentation and anti-inflammatory mediators production (10). However, deficiencies in apoptotic cell removal promote the turning of apoptotic cells into secondary necrotic cells, whereby they lose their membrane integrity and release danger signals, thus promoting inflammation and contributing to the initiation of autoimmunity (11, 12). In fact, accumulating evidence points to the relevant role of impaired efferocytosis in the development of multiple sclerosis, rheumatoid arthritis and T1D (13, 14).

Interestingly, the gold-standard experimental model of T1D, the non-obese diabetic (NOD) mouse, harbors a deficiency in the clearance of apoptotic cells by macrophages (15). In humans, polymorphisms in the *FCGR2A* gene, which encodes for the low-affinity IgG Fc region receptor II-a involved in phagocytosis, contribute to its pathogenesis (16). However, no data regarding the phagocytic function of DCs in human T1D are available. In patients with type 2 diabetes, there is growing evidence that impaired phagocytosis in neutrophils (17, 18) and macrophages (19) is related to glycemic control, albeit it can be alleviated with improved metabolic regulation. These data suggest that changes in glucose homeostasis can alter phagocytic processes, with the particularity that impairment of efferocytosis can contribute to the perpetuation of the autoreactivity in T1D.

By exploiting the inherent ability of apoptotic cell clearance to induce tolerance, a synthetic lipid-vesicle strategy that mimics apoptotic beta cells by being enriched in PS and encapsulating insulin was designed (20). This immunotherapy arrested autoimmunity upon administration to NOD mice after PS-liposomes were phagocyted by DCs, thus eliciting tolerogenic features. This finding was replicated in human DCs from adult patients with T1D (21). Therefore, this strategy achieves successful apoptotic mimicry and constitutes a promising strategy for tackling autoimmune diseases.

Unfortunately, T1D incidence is dramatically increasing in children (22, 23), in whom the disease is more aggressive and entails additional management complications. Since severe dysglycemia could impair DCs functionality, the aim of this study was to analyze circulating DCs subpopulations in pediatric T1D at different stages, as well as to characterize phagocytosis in T1D at the onset—hyperglycemic phase— and at established disease—when glucose levels are better controlled—, and to evaluate the role of phagocytosis in tolerance induction.

## Materials and Methods

### Patients and Control Individuals

Pediatric patients with T1D (*n* = 61) were visited as outpatients or hospitalized in the Pediatric Section at Germans Trias i Pujol Hospital. Patients were recruited at the onset and with a longer evolution (more than 6 months). Pediatric control individuals (*n* = 21) were also recruited. Inclusion criteria were 1–18 years of age and normal body mass index (BMI). Exclusion criteria were being under immunosuppressive or anti-inflammatory treatment, the presence of other autoimmune diseases, type 2 diabetes, pregnancy, and compromised kidney or liver function. All the experiments were carried out in strict accordance with the principles outlined in the Declaration of Helsinki for human research and after the approval of the Committee on the Ethics of Research of the Germans Trias i Pujol Hospital (PI-16-083). Signed informed consent was given by the parents of all subjects, and directly by children older than 12 years old.

### Analysis of Peripheral Blood DC Subsets

In order to analyze DCs subsets, 2 mL blood samples were collected in EDTA tubes from control subjects and patients with T1D at diagnosis, and at first and second year of disease progression. Blood samples were lysed (Lysing Buffer, BD Biosciences, San Jose, CA, USA) and stained with antibodies to CD45-AF700, CD3-APCH7, CD19-APCH7, CD14-V450, CD16-APC, CD11c-PECy7, CD123-PerCPCy5.5, CD56-PE, HLA-DR-V500, and Slan-FITC (BD Biosciences). A minimum of 10,000 events per sample was acquired using FACS LSRFortessa (BD Biosciences). Percentage and absolute counts (cells/μL) were analyzed using PerfectCount Microspheres (Cytognos SL, Salamanca, Spain) and FACSDiva software (BD Biosciences) following gating strategies based on international consensus (24).

### Human DC Generation

DCs were generated as described (21). Briefly, peripheral blood (10 mL) was obtained and monocytes were magnetically isolated using the StraightFrom Whole Blood CD14 MicroBeads kit (Miltenyi Biotech, Bergisch Gladbach, Germany). Monocytes were cultured at 10^6^ cells/mL in X-VIVO 15 media (Lonza, Basel, Switzerland), supplemented with 2% male AB human serum (Biowest, Nuaillé, France), 100 IU/mL penicillin (Normon SA, Madrid, Spain), 100 μg/mL streptomycin (Laboratorio Reig Jofré, Sant Joan Despí, Spain), and 1,000 IU/mL IL-4 and 1,000 IU/mL GM-CSF (Prospec, Rehovot, Israel). After 6 days, DCs differentiation yield was assessed by CD11c-APC staining (Immunotools, Friesoythe, Germany) and viability was determined by annexin V-PE (Immunotools) and 7-AAD (BD Biosciences) using FACSCanto II (BD Biosciences).

### Preparation of Liposomes

Liposomes consisted of 1,2-dioleoyl-sn-glycero-3-phospho-L-serine (sodium salt) (Lipoid, Steinhausen, Switzerland), 1,2-didodecanoyl-sn-glycero-3-phosphocholine (Lipoid), and cholesterol (Sigma-Aldrich). Liposomes were prepared using the thin-film hydration method from a lipid mixture of 1,2-dioleoyl-sn-glycero-3-phospho-L-serine, 1,2-didodecanoyl-sn-glycero-3-phosphocholine and cholesterol at 1:1:1.33 molar ratio, respectively, as described (20). Particle diameter and surface charge (ζ-potential) were measured by dynamic light scattering using Malvern Zetasizer (Malvern Instruments, Malvern, United Kingdom). Fluorescent liposomes (PSOG488-liposomes) were generated adding lipid-conjugated fluorescent dye Oregon Green 488 (OG488) 1,2-dihexadecanoyl-sn-glycero-3-phosphoethanolamine (ThermoFisher Scientific, Waltham, MA, USA) and had a lipid concentration of 30 mmol/L, a diameter size of 836 ± 217 nm (mean±SD), and a ζ-potential of −38.90 ± 2.52 mV. PS-liposomes encapsulating human insulin chains A (PSA-liposomes) and B (PSB-liposomes), were prepared as described (21) with the following physicochemical features: PSA-liposomes were 30 mmol/L of lipid concentration, 783 ± 104 nm of diameter and −38.15 ± 1.20 mV of ζ-potential; PSB-liposomes had 30 mmol/L of lipid concentration, 1040 ± 71 nm of diameter and −38.05 ± 0.07 mV of ζ-potential.

### Phagocytosis Assay

To determine whether DCs from patients with T1D had a defect in phagocytosis in the context of apoptotic cell removal, we tested DCs for their ability to capture synthetic PS-liposomes that mimic apoptotic beta cells. Then, DCs from control subjects, recent-onset patients and patients with established disease were cultured with 100 μmol/L PSOG488-liposomes at 37°C from 5 min to 6 h. As a negative control, the same assay was performed at 4°C to confirm that liposomes were captured by phagocytosis. DCs were extensively washed in cold PBS to remove liposomes attached to the cell membrane. Liposome capture was assessed by flow cytometry (FACSCanto II, BD Biosciences).

### Gene Expression Analysis

To determine gene expression changes after phagocytosis, DCs were cultured in basal conditions (immature DCs, iDCs) and with PS-liposomes encapsulating human insulin chains A (PSA-liposomes) and B (PSB-liposomes), as described (21). A mixture of PSA-liposomes and PSB-liposomes (PSAB-liposomes, 1 mmol/L) were simultaneously cocultured with DCs from control subjects, recent-onset patients and patients with established disease at 37°C for 4 h to promote tolerogenic features (tolerogenic DCs, tolDCs). RNA was isolated (RNeasy Micro Kit, QIAGEN, Hilden, Germany) and reverse-transcribed (High Capacity cDNA Reverse Transcription Kit, ThermoFisher Scientific). cDNA synthesis reactions were performed using random hexamers (0.5 mg/mL, BioTools, Valle de Tobalina, Madrid, Spain) and reverse transcriptase Moloney-murine-Leukemia-virus (200 U/mL, Promega, Madison, WI, USA). Targeted cDNA was pre-amplified with TaqMan PreAmp Master Mix (ThermoFisher Scientific), and qPCR was performed with TaqMan universal assays (ThermoFisher Scientific) on a LightCycler® 480 (Roche, Mannheim, Germany) using the following ones: *CD36* (Hs00354519_m1), *CD68* (Hs00154355_m1), *CD274* (*PD-L1*) (Hs00204257_m1)*, IDO1* (Hs00984148_m1)*, IL10* (Hs00961622_m1)*, LAIR1* (Hs00253790_m1), *MERTK* (Hs01031979_m1), *MFGE8* (Hs00983890_m1), *PDCD1LG2* (*PD-L2*) (Hs00228839_m1)*, PPARG* (Hs01115513_m1), *TGFB1* (Hs00998133_m1), *TNFAIP3* (Hs00234713_m1), *TNFSF14* (Hs00542476_g1), and *VEGFA* (Hs00900055_m1). Relative quantification was performed by normalizing the expression of each gene to that of the reference gene *GAPDH* (Hs02758991_g1), as described in the 2^−Δ*Ct*^ method (25).

### DCs Phenotype After Phagocytosis

DCs from patients at onset and with established disease were cultured for 24 h with 20 μg/mL human insulin (Sigma-Aldrich, St. Louis, MO, USA) and 1 mmol/L of PSAB-liposomes to determine the effect of insulin chains as autoantigens (tolDCs). As controls, DCs were either cultured with 20 μg/mL human insulin (Sigma-Aldrich) to obtain iDCs or adding a cytokine cocktail —TNF-α (1,000 IU/mL, Immunotools), IL-1β (2,000 IU/mL, Immunotools), and Prostaglandin E_2_ (PGE_2_, 1 μmol/L, Cayman Chemical, Ann Arbor, MI, USA)— for 24 h to obtain mature DCs (mDCs). Viability and phenotype were analyzed by flow cytometry (FACSCanto II, BD Biosciences). DCs were stained with 7-AAD (BD Biosciences) and antibodies to CD11c-APC, CD86-FITC, HLA-ABC-FITC, HLA-DR-FITC, CD14-PE and CD40-APC (Immunotools), CD36-APCCy7, TIM4-APC, αvβ5 integrin-PE, CD54-PECy7, TLR2-FITC, CXCR4-APCCy7, CCR2-APC (BioLegend, San Diego, CA, USA). Data were analyzed using FlowJo software (Tree Star Inc, Ashland, OR, USA).

### Cytokine Production

The Human Th1/Th2/Th17 kit (CBA system; BD Biosciences) was used to measure cytokine production. Culture supernatants from DCs (n≥3 for control subjects and n≥3 for patients with T1D) were collected and frozen at −80°C until use. IL-2, IL-4, IL-6, IFN-γ, TNF-α, IL-17A, and IL-10 were assessed. Data were analyzed using CBA software. VEGFA secretion by DCs was determined in culture supernatant by ELISA (ThermoFisher Scientific).

### Statistical Analysis

Prism 7.0 software (GraphPad Software Inc, San Diego, CA, USA) was used for the statistical analysis. For comparisons of unpaired data, a non-parametric Mann-Whitney test was used; for paired comparisons, the non-parametric Wilcoxon test was used. ANOVA was used for comparisons with several factors. For correlation between parameters, Spearman's test was used. A *p* value < 0.05 was considered significant.

## Results

### DCs Subsets From Peripheral Blood Are Altered at the Onset and Early Stages of T1D Progression

As a first approach, different subsets of circulating DCs were assessed in control subjects and patients with T1D at diagnosis, and at first year and second year of progression. Their clinical data are summarized in Table 1. All subjects met the inclusion and exclusion criteria and gave informed consent. Aside from differences in the evolution of the disease, patients at onset showed higher glycated hemoglobin (HbA_1c_) levels than patients at first year and at second year of progression. Patients at second year of progression required higher insulin doses than patients at onset and at first year of evolution.

**Table 1:** Data from the patients with T1D and control subjects included in the peripheral blood DCs subsets analysis.

**Parameter**	**Control subjects**	**Patients at onset**	**Patients at** **first year** **of evolution**	**Patients at** **second year** **of evolution**
*n*	10	11	11	6
Gender F/M	6/4 (60%/40%)	6/5 (55%/45%)	8/3 (73%/27%)	5/1 (83%/17%)
Age (years)	7.36 ± 2.52	8.35 ± 5.92	10.73 ± 3.73	10.02 ± 2.78
BMI (kg/m^2^)	17.42 ± 2.19	17.19 ± 3.05	17.94 ± 2.59	18.13 ± 2.23
Age at diagnosis (years)	NA	8.35 ± 5.92	10.17 ± 3.70	8.45 ± 2.73
Progression (years)	NA	0.00 ± 0.00	0.56 ± 0.18^****^	1.57 ± 0.10^****^/^++++^
HbA_1c_ (mmol/mol)	NP	92.05 ± 22.36	55.79 ± 10.78^****^	67.21 ± 12.76*
HbA_1c_ (%)	NP	10.57 ± 2.05	7.25 ± 0.99^****^	8.30 ± 1.17*
Fasting C-peptide (ng/mL)	NP	0.40 ± 0.40	<0.1 in 6/11 (55%) patients 0.72 ± 0.90	<0.1 in 3/6 (50%) patients 0.27 ± 0.06
Stimulated C-peptide (ng/mL)	NP	0.98 ± 1.02	NP	NP
Insulin dose (IU/kg/day)	NA	0.70 ± 0.19	0.60 ± 0.24	0.96 ± 0.18*/^++^

Data presented as mean ± SD; p value calculated from Mann-Whitney test (*p < 0.05, ****p < 0.0001 when comparing pediatric patients at onset group with pediatric patients at first or second year of evolution; ^++^p < 0.01, ^++++^p < 0.0001 when comparing pediatric patients at first year of evolution with those at second year of evolution).

*F, female; M, male; BMI, Body Mass Index; NA, Not Applicable; HbA_1c_, glycated hemoglobin; NP, Not Performed*.

**Figure 1:**
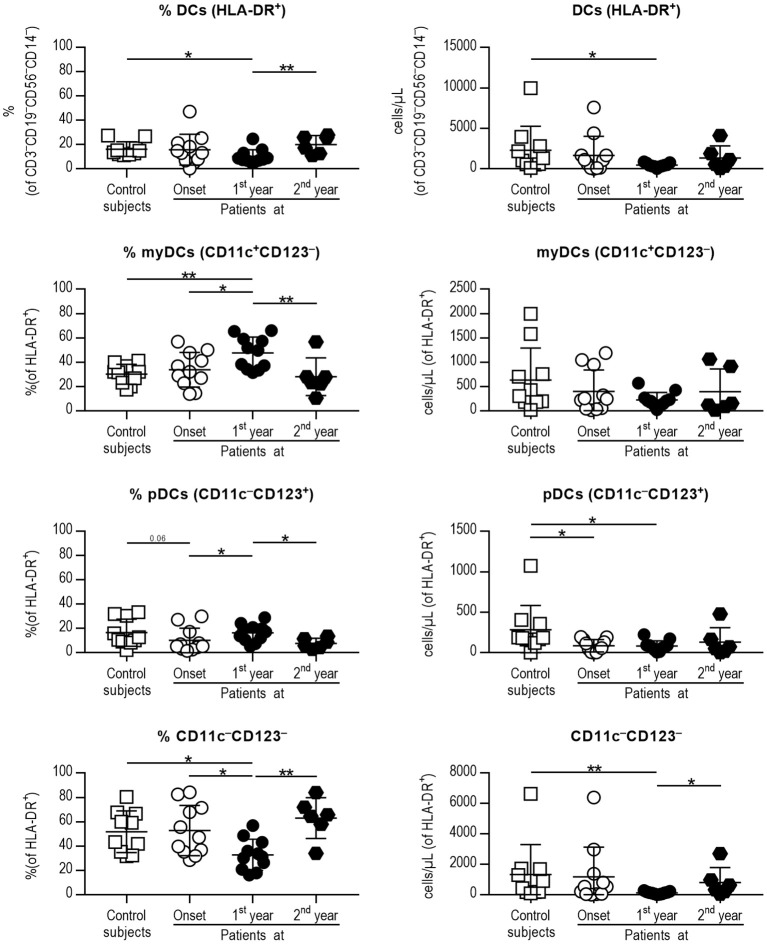
Peripheral blood DC subsets are quantitatively altered at the early stages of T1D. Percentages (left panel) and concentration (cells/μL, right panel) of total DCs (1st row, HLA-DR^+^ cells of CD3^−^CD19^−^CD56^−^CD14^−^ cells), myeloid DCs (myDCs, 2nd row, CD11c^+^CD123^−^ cells of HLA-DR^+^ cells), plasmacytoid DCs (pDCs, 3rd row, CD11c^−^CD123^+^ of HLA-DR^+^ cells) and the CD11c^−^CD123^−^ subset (4th row, CD11c^−^CD123^−^ of HLA-DR^+^ cells). Data are expressed as mean ± SD; white squares are control subjects (*n* = 10), white circles are patients at onset (*n* = 11), black dots are patients at first year of progression (*n* = 11), and black rhombuses are patients at second year of progression (*n* = 6). Statistically significant differences were found when comparing the groups (*p < 0.05, **p < 0.01, Mann-Whitney test).

Thus, four different subsets of DCs in peripheral blood [total DCs, myeloid DCs (myDCs), plasmacytoid DCs (pDCs) and a CD11c^−^CD123^−^ putative DC subset] were analyzed (Figure 1) following the gating strategy displayed in Figure S1. As for total DCs, percentages and numbers were decreased in patients at first year when compared to control subjects, although patients at second year recovered a normal percentage. The percentage of myDCs was increased in patients at first year when compared to control subjects, patients at onset and at second year of progression, although no differences were observed in their numbers. As for pDCs, patients at first year displayed a higher percentage than patients at onset and patients at second year. The numbers of pDCs were higher in control subjects when compared to patients at onset and at first year. Finally, patients at first year had a decreased percentage of the CD11c^−^CD123^−^ subset when compared to control subjects, patients at onset and at second year, and their numbers were also lower when compared to control subjects and patients at second year. Therefore, DCs subsets from peripheral blood were found quantitatively altered during the first year of childhood T1D and recovered in the second year of progression.

### DC Differentiation Efficiency Is Similar in Pediatric Patients and Control Subjects

After observing alterations in DCs subsets in pediatric patients with T1D, and in order to assess their functionality, DCs were derived from peripheral blood monocytes from patients at diagnosis and with established disease as well as from control subjects. Their clinical data are summarized in Table 2. The BMI of patients with established disease was significantly higher than that of control subjects and patients at onset. As expected, patients with established disease had a longer evolution and significantly lower HbA_1c_ values than patients at onset. Then, monocytes were magnetically isolated directly from whole blood, and differentiated into DCs *in vitro*. The number of isolated CD14^+^ cells (monocytes), viability, and dynamics of the CD11c marker acquisition (differentiation to DCs) at days 1, 4, and 6 of culture showed similar values in the three groups (Table 3).

**Table 2:** Main clinical characteristics and metabolic data of participants included in the study of phagocytosis and tolerance induction.

**Parameter**	**Control** **subjects**	**Patients** **at onset**	**Patients with** **established disease**
*n*	11	14	19
Gender F/M	8/3 (73%/27%)	9/5 (64%/36%)	12/7 (63%/37%)
Age (years)	9.56 ± 1.66	9.74 ± 3.54	12.13 ± 3.92
BMI (kg/m^2^)	17.50 ± 2.61	16.57 ± 3.08	20.33 ± 3.80^*^/^++^
Age at diagnosis (years)	NA	9.74 ± 3.54	8.46 ± 3.28
Progression (years)	NA	0.00 ± 0.00	3.66 ± 3.00^++++^ (range: 0.5–11.7 years)
HbA_1c_ (mmol/mol)	NP	113.50 ± 20.92	61.92 ± 8.75^++++^
HbA_1c_ (%)	NP	12.54 ± 1.92	7.82 ± 0.80^++++^
Fasting C-peptide (ng/mL)	NP	0.46 ± 0.28	<0.1 in 13/19 patients (68%) 0.4 ± 0.32
Stimulated C-peptide (ng/mL)	NP	0.88 ± 0.58	NP
Insulin dose (IU/kg/day)	NA	0.85 ± 0.22	0.89 ± 0.17

*Data presented as mean ± SD*.

p value calculated from Mann-Whitney test (*p < 0.05 when comparing either group of patients to the control group; ^++^p < 0.01, ^++++^p < 0.0001 when comparing patients at onset with those with established disease).

*F, female; M, male; BMI, Body Mass Index; NA, Not Applicable; HbA_1c_, glycated hemoglobin; NP, Not Performed*.

**Table 3:** Data from monocyte isolation and DC differentiation.

**Parameter**	**Control** **subjects**	**Patients at** **onset**	**Patients with** **established disease**
*n*	11	14	19
Isolated CD14^+^ cells (day 1, × 10^6^ cells)	2.60 ± 0.84	2.83 ± 1.46	2.57 ± 1.07
Viability (day 1, %)	96.13 ± 2.34	96.29 ± 3.33	97.16 ± 1.87
CD11c purity (day 1, %)	4.23 ± 3.24	4.56 ± 2.68	5.41 ± 4.87
Differentiation efficiency (CD11c purity, day 4, %)	63.50 ± 18.59	61.46 ± 25.27	71.74 ± 25.79
Differentiation efficiency (CD11c purity, day 6, %)	92.17 ± 6.06	88.31 ± 9.19	86.95 ± 13.08

*Data presented as mean ± SD. No statistical differences were found between groups (Mann-Whitney test)*.

### DCs From Patients With T1D Display Impaired Phagocytic Activity in Correlation With Disease Progression

**Figure 2:**
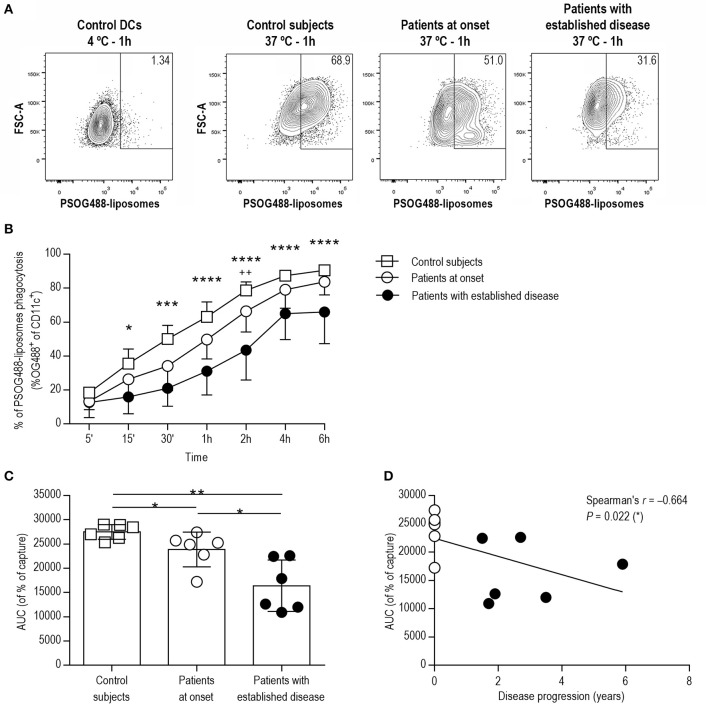
DCs from patients with T1D show impaired phagocytic activity in correlation with the time of disease progression. **(A)** Flow cytometry contour plots and percentages of the capture of PSOG488-liposomes by DCs (CD11c^+^). From left to right, control DCs cultured with PSOG488-liposomes at 4°C for 1 h, and DCs from control subjects, patients at onset and patients with established disease cultured with PSOG488-liposomes at 37°C for 1 h. One representative experiment of 6 is shown. **(B)** Time-course of the capture of PSOG488-liposomes by DCs obtained from control subjects (white squares, *n* = 6), pediatric patients at onset (white circles, *n* = 6) and with established disease (black dots, *n* = 6) at 37°C. Results are mean±SEM. Comparisons between groups showed significant differences (*p < 0.05, ***p < 0.001, ****p < 0.0001 when comparing control subjects with patients with established disease; ^++^*p* < 0.01 when comparing patients at onset with patients with established disease, Two-way ANOVA). **(C)** Area under the curve (AUC) of phagocytosis kinetics curves. Differences were found between groups (*p < 0.05, **p < 0.01, Mann-Whitney test). **(D)** Correlation between AUC values of phagocytosis kinetics curves and disease progression. A significant correlation was found between both parameters (Spearman's *r* = −0.664, *p* = 0.022, Spearman's correlation analysis).

In order to test the ability of DCs to phagocyte lipid vesicles in the context of apoptotic cell removal, fluorescent PS-liposomes were used in a time-course experiment, coculturing PSOG488-liposomes with DCs from each group of subjects. As expected, phagocytosis was not observed at 4°C (Figure 2A). At 37°C (Figures 2A,B), the phagocytosis rate of PSOG488-liposomes by DCs from patients with established disease was decreased when compared to control subjects, starting at 15 min of coculture and throughout the assay. Moreover, DCs from patients with established disease had phagocyted lower levels of PSOG488-liposomes than patients at onset at 2 h of coculture. No significant differences were found when comparing the phagocytosis kinetics of DCs from patients at onset and control subjects. To further dissect this finding, the area under the curve (AUC) of the kinetics of capture was calculated (Figure 2C). The AUC values of patients with established disease were significantly lower than those of patients at onset and control subjects. Also, patients at onset had significantly lower AUC values than control subjects. Therefore, the correlation between AUC values and the time of disease progression was analyzed (Figure 2D), discovering that both parameters were significantly and inversely correlated (Spearman's *r* = −0.664, *p* = 0.022). This defect was only observed in children with T1D, and not in adults, as previously reported (21). Thus, and as an additional control, we have included in this study a phagocytosis assay with DCs from adult patients with established disease, which demonstrates that the impairment of phagocytosis is an inherent feature of pediatric T1D (Figure S2 and Table S1).

### Efferocytosis and Immunoregulation-Related Gene Expression Is Altered in DCs From Patients With T1D

**Figure 3:**
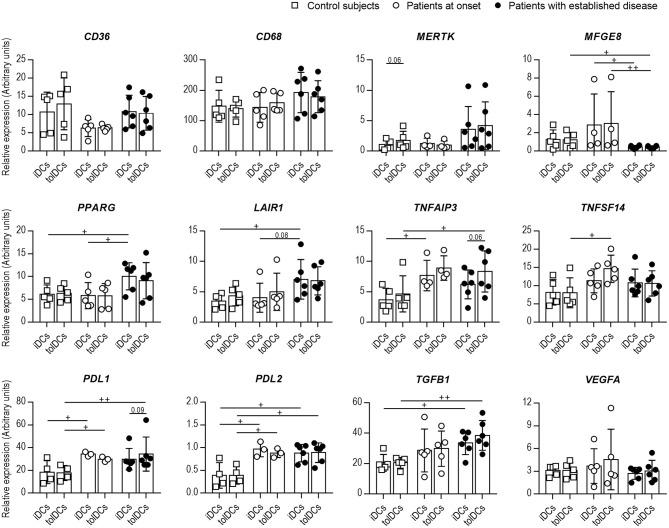
The expression of genes related to efferocytosis and immunoregulation is altered in DCs from patients with T1D. Expression of *CD36, CD68, MERTK, MFGE8, PPARG, LAIR1, TNFAIP3, TNFSF14, PDL1, PDL2, TGFB1*, and *VEGFA* genes in immature DCs (iDCs) before and 4 h after (tolDCs) coculture with PSAB-liposomes, analyzed by qPCR. White squares are control subjects (*n* = 5), white circles are patients at onset (*n* = 3-5), and black dots are patients with established disease (*n* = 6). Bars show the mean ± SD. Gene expression signals were normalized to *GAPDH* expression. No significant differences were found when comparing culture conditions in the same group (Wilcoxon test). Significant differences were found when comparing the same culture conditions in the three groups (^+^*p* < 0.05, ^++^*p* < 0.01, Mann-Whitney test).

Since the phagocytic capacity of DCs from patients with T1D was altered, the gene expression of specific PS receptors (*CD36, CD68, MERTK, MFGE8*), and immunoregulatory molecules (*PPARG, TNFAIP3, TNFSF14, LAIR1, PDL1, PDL2, TFGB1*, and *VEGFA*) was evaluated in DCs before (iDCs) and 4 h after (tolDCs) coculture with PSAB-liposomes in the three groups: control subjects, patients at onset and patients with established disease (Figure 3). All these genes were expressed in iDCs. Firstly, no significant differences in the expression of *CD36, CD68* nor *MERTK* were found between groups, but a significant reduction in *MFGE8* expression was detected in DCs from patients with established disease in comparison to the other groups. Moreover, after liposome capture, the expression levels of genes of PS receptors were preserved or even showed a tendency to increase in comparison to iDCs. In this sense, a tendency for upregulation of *MERTK —*a feature of tolDCs (26)— was observed in tolDCs from control subjects (*p* = 0.06).

Regarding genes of immunoregulatory molecules, *PPARG* and *LAIR1* expression discerned both stages of T1D, being higher in established disease than at the onset and controls. Patients with T1D also showed higher levels of *TNFAIP3, TNFSF14, PDL1, PDL2, and TGFB1* in DCs than controls. Interestingly, tolDCs from patients exhibited a trend for upregulation of the *TNFAIP3, TNFSF14*, and *TGFB1* genes upon PSAB-liposomes capture at different stages of the disease. Finally, *VEGFA* gene expression did not differ between groups nor after the capture of PSAB-liposomes. Concerning *IDO1* and *IL10*, a low expression was detected in all conditions (Figure S3).

### DCs From Pediatric Patients Acquire a Tolerogenic Potential After Phagocytosis of PS-liposomes

**Figure 4:**
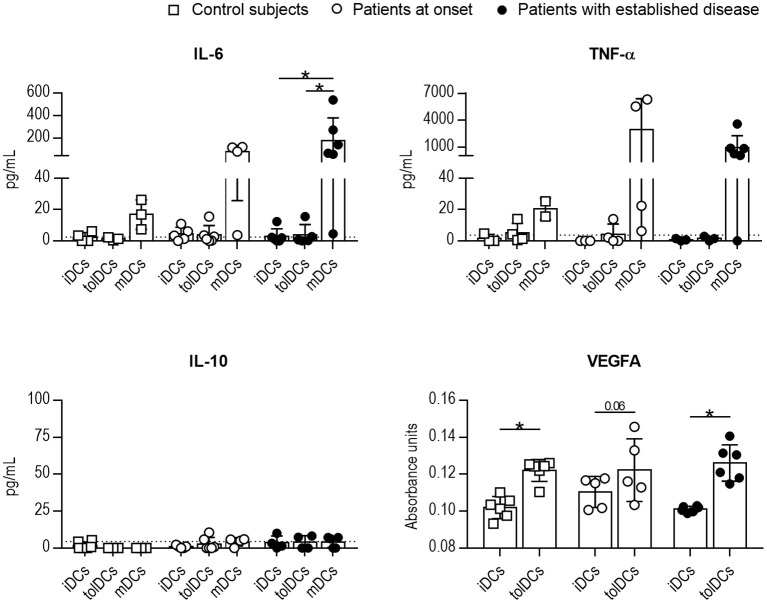
The cytokine profile after the uptake of PSAB-liposomes by DCs points to tolerogenic potential. Concentration of IL-6, TNF-α, IL-10, and VEGFA secreted by DCs obtained from control subjects (white squares, *n* ≥ 3), patients with T1D at onset (white circles, *n* ≥ 3) and patients with established disease (black dots, *n* ≥ 3). Bars represent immature DCs (iDCs), immature DCs 4 h after the capture of PSA-liposomes and PSB-liposomes (tolDCs), or mature DCs (mDCs) induced with a cytokine cocktail. Data presented as mean ± SD. Significant differences were found when comparing the different conditions in the same group of subjects (*p < 0.05, Wilcoxon test). Differences were not found when comparing the same culture condition in the three groups (Mann-Whitney test).

In terms of cytokine secretion by DCs, the release of IL-6, TNF-α, IL-10, and VEGFA by DCs from control subjects, recent-onset patients and patients with established disease was consistent with a tolerogenic profile after PSAB-liposomes phagocytosis (Figure 4). Briefly, IL-6 concentration was similar before (iDCs) and after (tolDCs) PSAB-liposomes uptake in all groups. Moreover, IL-6 secretion increased after maturation stimulus in DCs (mDCs) from patients with established T1D, and the same trend was observed in the other groups. Similarly, TNF-α was not increased after liposome uptake, and its secretion tended to increase in pro-inflammatory conditions. IL-10 concentration was detected around the limit of detection in all samples. Remarkably, VEGFA concentration was slightly increased in the supernatant of DCs after liposome uptake when compared to that of iDCs in control subjects and patients with established disease —although patients at onset showed the same tendency (*p* = 0.06). IL-2, IL-17A, and IFN-γ were not detected in any condition of the assay (data below the detection limit). These results, although not always statistically significant, support the ability of DCs from children with T1D to achieve a tolerogenic phenotype.

**Figure 5:**
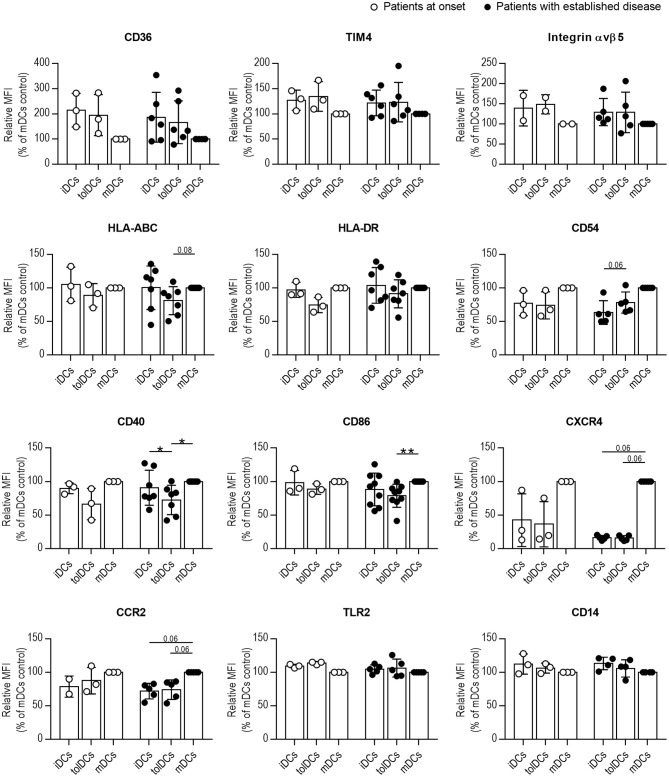
Phagocytosis of PS-liposomes induces a tolerogenic phenotype in DCs from pediatric patients both at onset and established T1D. Membrane expression of CD36, TIM4, Integrin αvβ5, HLA-ABC, HLA-DR, CD54, CD40, CD86, CXCR4, CCR2, TLR2, and CD14 molecules in DCs obtained from patients at onset (white circles, *n* = 2–3) and with established disease (black dots, *n* = 4–9). Bars represent immature DCs (iDCs), iDCs after the capture of PSAB-liposomes (tolDCs), and mature DCs (mDCs) induced with a cytokine cocktail, after 24 h. Data are mean ± SD of relative Median of Fluorescence Intensity (MFI), this being MFI of each condition referred to their respective mDCs control. Significant differences were found when comparing culture conditions in the same group (*p < 0.05, **p < 0.01, Wilcoxon test), but not when comparing the same culture conditions in both groups (Mann-Whitney test).

Afterward, we interrogated whether the phenotype of DCs from patients changed toward a tolerogenic profile after 24 h of coculture with PSAB-liposomes (tolDCs), despite the phagocytosis defect. First, the viability of DCs from all groups was higher than 90% in all conditions, thus confirming that PS-liposomes are not toxic to DCs obtained from pediatric subjects (data not shown). No differences were observed in the DCs phenotype between patients at onset or with established disease, under any of the conditions (Figure 5). Membrane expression of PS receptors such as CD36, TIM4 and Integrin αvβ5 was not altered after liposome capture in comparison to iDCs. Then, the expression of molecules related to the immune function of DCs was assessed. Antigen-presenting molecules HLA-ABC displayed a trend for lower expression in tolDCs when compared to mDCs in patients with established disease (*p* = 0.08); HLA-DR expression did not exhibit any changes. As for adhesion molecules such as CD54, in patients with established disease, tolDCs showed a tendency for higher expression than iDCs (*p* = 0.06). Regarding costimulatory molecules CD40 and CD86, lower expression was observed in tolDCs when compared to mDCs in patients with established disease, and patients at onset displayed the same pattern. Chemokine receptors CXCR4 and CCR2 were not upregulated after phagocytosis; however, mDCs from patients with established disease tended to express higher levels of these molecules when compared to iDCs and tolDCs (*p* = 0.06). Finally, no defects in the expression of pattern recognition receptors, TLR2 and CD14, were observed in DCs under any conditions.

## Discussion

It is well-known that DCs, as orchestrators of the immune response with a crucial role in maintaining self-tolerance, are highly involved in the pathogenesis of T1D. Several studies have enumerated the broad spectrum of alterations that DCs from these patients have, ranging from genetic alterations, to phenotypic and functional divergences (16). Fewer studies, however, have focused on the potential of DCs from adult patients to become tolerogenic (27, 28), but no data are available in pediatric patients. To our knowledge, this is the first work that assesses DCs subsets in children with T1D at different stages of the disease and evaluates their tolerogenic potential. Indeed, we believe these are paramount aspects to consider in the near future, when antigen-specific immunotherapies will be available and immunomonitoring of the patients will be required.

Thus, peripheral blood DC subsets were measured to determine quantitative alterations related to disease progression. Interestingly, most of the differences gathered in the first year of evolution were restored in the second year. Briefly, the percentage of myDCs and pDCs was increased at diagnosis when compared to the other time points, whereas the CD11c^−^CD123^−^ subset was decreased. In this sense, alterations in myDCs and pDCs subsets have been reported in children with T1D at initial stages of disease (29–31), and further efforts are warranted to validate the usefulness of DC subsets as biomarkers in the progression of the disease and to provide further insight into their biological significance.

The alteration in DCs subsets at the early stages of T1D was an early observation that prompted us to study the tolerogenic functionality of DCs. To do so, we employed a nanotherapy imitating apoptotic beta cells consisting of PS-liposomes encapsulating insulin (20). These liposomes were phagocyted by murine DCs, thus arresting autoimmunity in the experimental model of the disease, and also induced tolerogenic features in human DCs from adult patients with T1D (21).

In the present study, three similar groups of pediatric subjects in terms of gender and age were included. Patients with established disease had higher BMI, which was expected according to the recovery of weight after disease onset and to the distribution of BMI related to age (32), but nonetheless healthy, in order to avoid the hypothetical interference of inflammatory processes associated with overweight and obesity. Quite unexpectedly, here we found that the phagocytic activity of monocyte-derived DCs from pediatric patients with T1D was impaired, and this defect deteriorated with the progression of the disease but did not affect the induction of a tolerogenic phenotype after phagocytosis. This immune dysfunction, not previously reported in pediatric patients with T1D, agrees with studies that have reported an impaired phagocytic activity in macrophages from the NOD mouse, both at early ages—before diabetes development—and at adulthood when compared to other strains (15). An attenuated phagocytic activity has also been found in patients with type 2 diabetes, but it was reversed after the improvement of glycemic control (19). Moreover, a reduced capacity for efferocytosis has been reported in other autoimmune diseases unrelated to glucose metabolism (13). In addition, specific PS receptors are crucial in efferocytosis. As expected, mice deficient in PS receptors show impaired efferocytosis and increased inflammation (11). Overall, these data suggest that several factors, e.g., intrinsic defects, dysglycemia and inflammation, may contribute to the impairment of phagocytosis in T1D. It is well-known that efferocytosis is not only a mechanism of apoptotic cell removal but also a crucial process in the maintenance of immunological homeostasis, and so this defect could contribute to the persistent lack of self-tolerance.

Although DCs from pediatric patients with T1D were found to have impaired phagocytosis in correlation with disease progression, DCs from patients at onset and with established disease showed similar expression of PS receptors (*CD36, CD68*, and *MERTK*), except for *MFGE8*. Interestingly, DCs from patients with established disease had a decreased *MFGE8* expression, and this defect could contribute to the herein reported phagocytosis impairment. Since several PS receptors work to internalize apoptotic cells (33), the suboptimal expression or function of any of them could explain the altered phagocytosis. In fact, scarce information has been reported about DCs phagocytic activity, since most studies have been conducted with macrophages. Thus, it is plausible that DCs may express the same PS receptors as macrophages but in different proportions, or express entirely different PS receptors (34), which could be accountable for the impaired phagocytosis of DCs.

As for the immunoregulatory profile, DCs from pediatric patients with T1D showed higher expression of *PPARG* and *TGFB1*—two genes downstream in the efferocytosis signaling pathway—than control subjects. Moreover, *TNFAIP3* displayed higher expression in DCs from patients; interestingly, the expression of *Tnfaip3* in murine DCs was reportedly upregulated after efferocytosis (35), thus reducing their rate of apoptotic cell clearance and prompting their acquisition of tolerogenic potential (36). Also, the overexpression of *LAIR1*—detected in DCs from patients with established disease—has been linked to an inhibition of cholesterol-particle phagocytosis by murine macrophages (37). Thus, the expression of these two genes combined could also provide a plausible explanation for the observed impaired phagocytosis of PS-liposomes. In addition, their higher expression in comparison to control subjects, along with that of *PPARG, PDL1, PDL2*, and *TGFB1* genes, could reflect a continuous attempt of immunoregulation prompted by the ongoing autoimmune reaction (38). Importantly, the expression of tolerogenic markers was also found upregulated in adult DCs after the capture of PSAB-liposomes in our previous RNA-seq analysis (21). However, other factors, such as IDO and IL-10, have been shown to inhibit T cell activation by DCs after efferocytosis, but we did not observe any increase in *IDO1* and *IL10* expression, in line with similar studies (39).

Moreover, the results of the characterization of the cytokine profile in DCs from pediatric patients were similar to those reported in adults (21), and support the ability of DCs from children with T1D to achieve a tolerogenic phenotype. As in adults, IL-10 was detected at very low levels in all conditions, correlating with the above-mentioned gene expression results, although this could be explained by the timing of the experiment. Nevertheless, it is worth mentioning the profile of VEGFA secretion—a cytokine involved in the inhibition of DCs maturation after apoptotic cell removal (40, 41)—, in which tolDCs from the three groups released higher levels of VEGFA in comparison to iDCs, 4 h after phagocytosis. This further supports that DCs acquire tolerogenic properties after PS-liposomes phagocytosis, even if their phagocytosis ability is impaired. Overall, these results suggest that DCs from patients could mediate immunoregulatory attempts.

Finally, membrane expression of PS receptors and immune-related molecules in DCs from pediatric patients with T1D was also studied to determine whether the activation of tolerogenic signaling pathways was translated to the DC membrane. These PS receptors are expressed in iDCs to allow apoptotic cell phagocytosis. As described previously (21), the expression of PS receptors was maintained after PSAB-liposomes phagocytosis or even showed a tendency to increase in comparison to iDCs, thus preserving the ability to efferocyte. As for the expression of immune-related molecules, tolDCs from pediatric patients, both at onset and with established disease, showed a pattern consistent with tolerogenic potential. Indeed, this phenotype is similar to that displayed by DCs after PS-liposomes uptake in murine models of T1D and multiple sclerosis (20, 42), and also in human DCs from adult patients with T1D (21). However, the main limitation in the studies of tolDCs is the lack of a robust single marker of tolerogenic potential, and so several parameters that altogether point to the tolerogenic potential of DCs are usually determined. To date, tolDCs are accepted as reluctant to maturation and stable in a state of low expression of antigen-presenting and costimulatory molecules, along with a tolerogenic-skewed cytokine profile (43). Thus, the here presented data are consistent with a biological tolerogenic effect in pediatric DCs after liposome uptake.

We are well-aware of the limitations of the study. On the one hand, efferocytosis of apoptotic beta cells was mimicked using previously-described synthetic microvesicles displaying features of apoptotic cells (20). However, the engulfment of these liposomes by DCs induces similar effects to those observed by physiological apoptotic cell phagocytosis and constitutes a good system to test the fitness of DCs. On the other hand, only six PS receptors were explored, and despite their crucial role in efferocytosis, other receptors could be altered in T1D thus impairing DCs phagocytosis. Whether this alteration is instrumental in the progression of the autoimmune attack toward the clinical manifestation of T1D is currently under investigation. In fact, when the clinical diagnosis of T1D takes place, there is already a significant loss of beta cells in the pancreas of the patient. However, in this scenario, autoimmunity to beta cells remains active despite the low numbers of alive or functional beta cells in the pancreatic islets, and it can destroy these remaining beta cells. Thus, the altered phagocytic activity observed in DCs could be translated into impaired phagocytosis of actual apoptotic beta cells, which could facilitate their conversion into secondary-necrotic cells if they are not efficiently removed by phagocytes. This happening would increase the availability of danger signals and feed the autoimmune process against beta cells. Perhaps, children susceptible to the disease may carry a defect in the efferocytosis ability of phagocytes, which could further exacerbate immunogenic responses. Interestingly, the here identified defect in the phagocytic activity of DCs from pediatric patients with T1D was not detected in adult patients (21), so we can speculate that childhood features (growth, puberty, environmental factors) and diabetes (metabolic control, immune response heterogeneity) could influence DCs behavior (44, 45). Nevertheless, the preservation of the tolerogenic potential in DCs from pediatric patients with T1D could help in restraining the autoimmune destruction of beta cells, and in preserving the residual beta cell mass in patients with T1D.

In conclusion, pediatric peripheral DCs subsets are altered in the first stages of T1D and their phagocytic capacity deteriorates with disease progression, although tolerance induction could still function optimally. Despite further studies to draw the intricate picture of this phagocytosis impairment are needed, both the study of peripheral blood DCs and functional DCs studies provide useful data in patient's follow up, and could aid in the stratification of patients in immunotherapy clinical trials, since these results could inform about the immunological status of the patients' DCs and their ability to promote a tolerogenic response.

## Data Availability Statement

All datasets generated for this study are included in the article/Supplementary Material.

## Ethics Statement

All the experiments were carried out in strict accordance with the principles outlined in the Declaration of Helsinki for human research and after the approval of the Committee on the Ethics of Research of the Germans Trias i Pujol Hospital. All subjects gave written informed consent in accordance with the Declaration of Helsinki.

## Author Contributions

SR-F, FV, and MV-P designed the experiments. MM, EA, FV, and JB selected the patients and control subjects and obtained clinical data. MC-S and DM generated the liposomes. SR-F, AV, DP-B, and LG-M performed the experiments. SR-F and MV-P wrote the manuscript. MM, AV, DP-B, LG-M, and FV reviewed the manuscript and contributed to the discussion. All authors revised the final manuscript and gave final approval of the version to be published.

### Conflict of Interest

MC-S, DM, and MV-P are co-founders of Ahead Therapeutics SL, which works towards the clinical translation of the PS-liposomes tolerogenic technology for the treatment of autoimmune diseases. The remaining authors declare that the research was conducted in the absence of any commercial or financial relationships that could be construed as a potential conflict of interest.

## Abbreviations

DCs: Dendritic Cells
iDCs: Immature Dendritic Cells
mDCs: Mature Dendritic Cells
myDCs: Myeloid Dendritic Cells
PBMCs: Peripheral Blood Mononuclear Cells
PS: Phosphatidylserine
pDCs: Plasmacytoid Dendritic Cells
TolDCs: Tolerogenic Dendritic Cells.
