# Supplementary material for: Impaired Phagocytosis in Dendritic Cells From Pediatric Patients With Type 1 Diabetes Does Not Hamper Their Tolerogenic Potential
Source: Front Immunol. 2019 Nov 28;10:2811. doi: 10.3389/fimmu.2019.02811 (PMC6892968; doi:10.3389/fimmu.2019.02811)
Supplement: Supplementary file 1 [file Data_Sheet_1.PDF]

SUPPLEMENTARY DATA

Impaired phagocytosis in dendritic cells from pediatric patients with type 1 diabetes does not hamper their tolerogenic potential

Running title: Dendritic cells in pediatric type 1 diabetes

Silvia Rodriguez-Fernandez[1], Marta Murillo[2], Adrian Villalba[1], David Perna-Barrull[1], Mary Cano-Sarabia[3], Laia Gomez-Muñoz[1], Eva Aguilera[4], Daniel Maspoch[3,5], Federico Vazquez[4], Joan Bel[2], Marta Vives-Pi[*1,6]

[1]Immunology Section, Germans Trias i Pujol Research Institute, Autonomous University of Barcelona, Badalona, Spain [2]Pediatrics Section, Germans Trias i Pujol University Hospital, Badalona, Spain [3]Catalan Institute of Nanoscience and Nanotechnology, CSIC and The Barcelona Institute of Science and Technology, Bellaterra, Spain [4]Endocrinology Section, Germans Trias i Pujol University Hospital, Badalona, [5]ICREA, Pg. Lluis Companys 23, Barcelona, Spain [6]CIBERDEM, ISCiii, Madrid, Spain-

[*]**Author for correspondence**: Marta Vives-Pi. Immunology Section. Germans Trias i Pujol Research Institute. Carretera Canyet s/n. 08916 Badalona, Spain. Phone +34934978666. e-mail, mvives@igtp.cat

SUPPLEMENTARY FIGURES

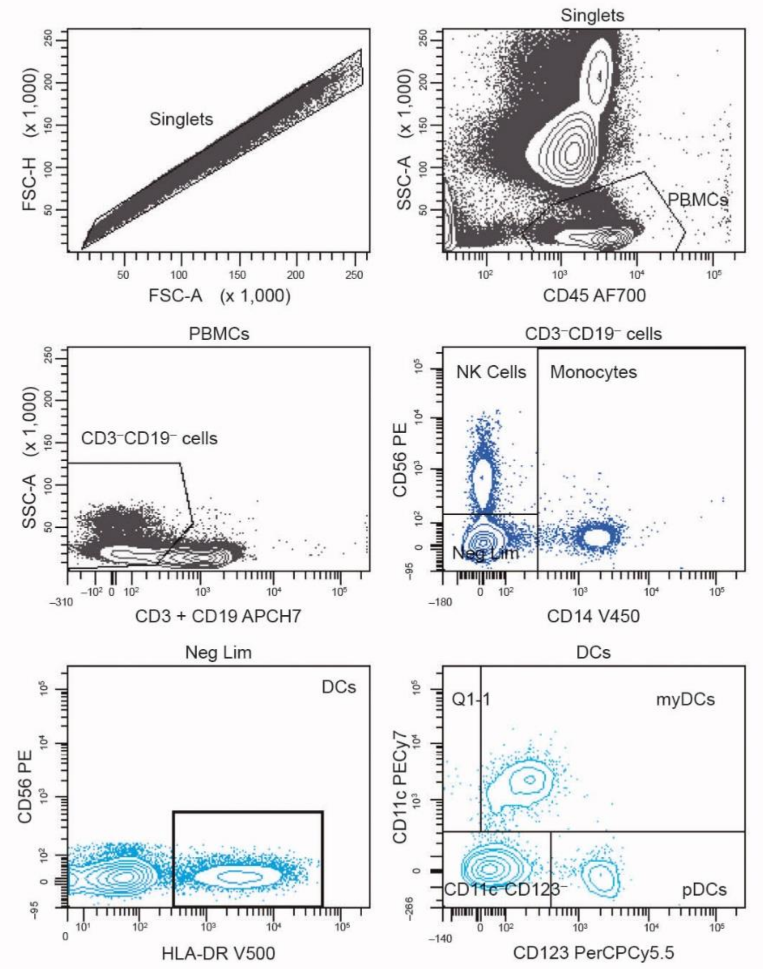

Population	#Events	%Parent	%Total
All Events	609,999	####	100.0
Singlets	485,841	79.6	79.6
PBMCs	78,534	16.2	12.9
CD3-CD19- cells	23,294	29.7	3.8
NK Cells	3,125	13.4	0.5
Neg Lim	18,109	77.7	3.0
DCs	4,067	22.5	0.7
Q1-1	24	0.6	0.0
myDC	932	22.9	0.2
CD11c-CD123-	2,647	65.1	0.4
pDC	464	11.4	0.1
Monocytes	2,060	8.8	0.3

Figure S1. Flow cytometry analysis of peripheral blood dendritic cells (DCs) subsets in pediatric subjects with type 1 diabetes. Gating strategy used to analyze the percentages and absolute counts of the different DCs subsets. Cells were first gated for singlets (FSC-A vs. FSC-H), and then peripheral blood mononuclear cells (PBMCs) were gated with the use of CD45-AF700 ($CD45^{+}$ vs. SSC-A). Then, T cells, B cells, NK cells and monocytes were excluded with the use of CD3-APCH7, CD19-APCH7, CD56-PE and CD14-V450, respectively. DCs were gated with the use of HLA-DR-V500. Finally, the different subsets of DCs were analyzed using CD123-PerCPCy5.5 and CD11c-PECy7, thus revealing 3 subsets: myeloid DCs (myDCs, $CD11c^{+}CD123^{-}$), plasmacytoid DCs (pDCs, $CD11c^{-}CD123^{+}$) and a non-categorized subset that is $CD11c^{-}CD123^{-}$. Shown is a representative gating strategy and contour plots of peripheral blood DCs obtained from a pediatric patient at the second year of evolution.

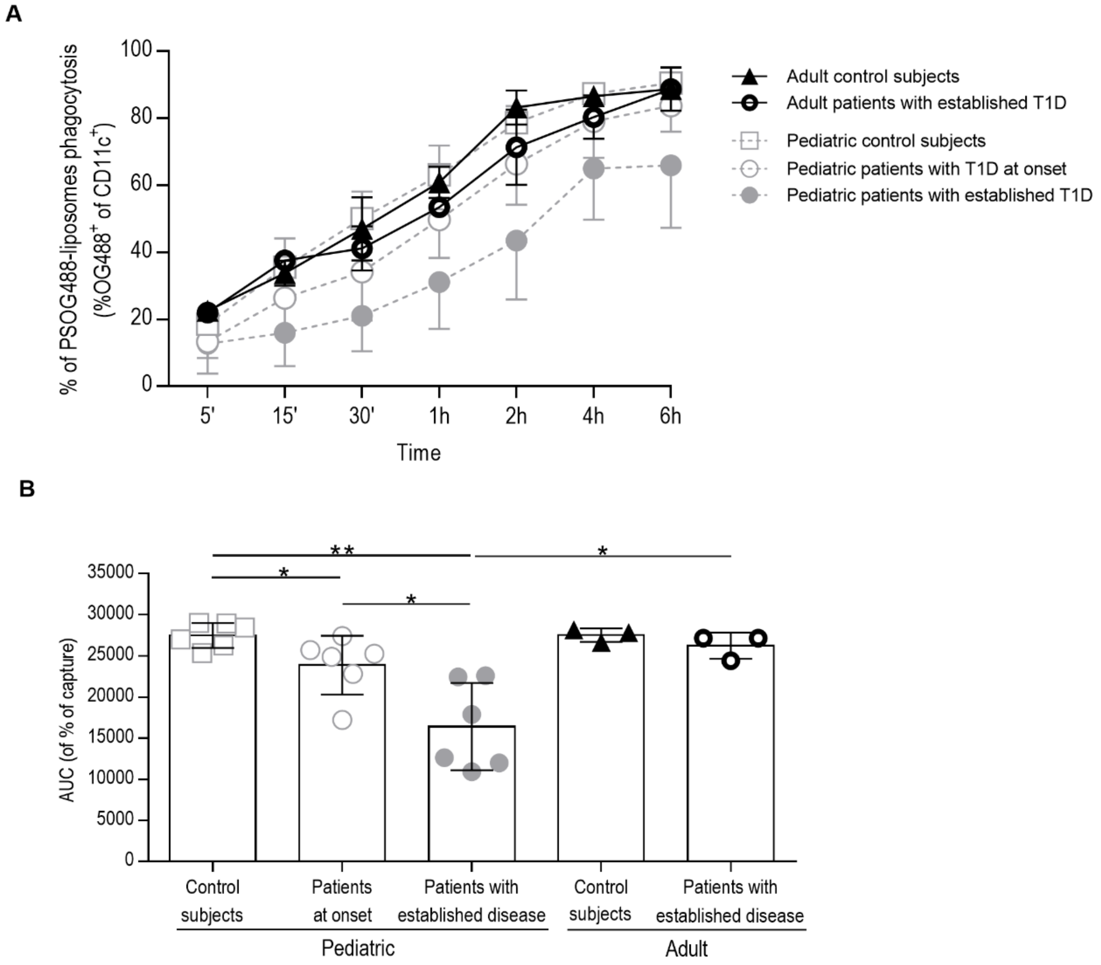

Figure S2. DCs from pediatric control subjects show a similar phagocytic activity of PSOG488-liposomes than DCs from adult control subjects and adult patients with established T1D. **(A)** Time course of the capture of PSOG488-liposomes by DCs obtained from adult control subjects (black triangles, n = 3), adult patients with established T1D (black circles, n = 3), pediatric control subjects (white squares, n = 6), pediatric patients at onset (white circles, n = 6) and with established disease (gray dots, n = 6) at 37 ºC. Results are mean±SEM. **(B)** AUC of phagocytosis kinetics curves. Differences were found between groups (*$p < 0.05$, **$p < 0.01$, Mann-Whitney test).

Supplementary Material for Figure S2

Adult patients and control individuals

Adult control individuals and patients with T1D were recruited to derive dendritic cells from peripheral blood monocytes, in order to compare the kinetics of PS-liposomes capture of pediatric DCs to adult DCs. The inclusion criteria for these subjects were 18-55 years of age, a BMI between 18.5 and 30 kg/m^2 and, for patients with T1D, an evolution of the disease longer than 6 months. Exclusion criteria were being under immunosuppressive or anti-inflammatory treatment or undergoing pregnancy or breastfeeding. All subjects met the inclusion and exclusion criteria and gave informed consent. Their clinical data is summarized in **Table S1** (see page 4 of this Supplementary

Data file). **Human DC generation** and **Phagocytosis Assay** were performed as described in the manuscript.

Table S1. Main clinical characteristics and metabolic data of the adult participants included in the study of phagocytosis

**Parameter**	**Adult control subjects**	**Adult patients with established T1D**
n	3	3
**Gender F/M**	1/2	3/0
**Age (years)**	27.0 ± 2.00	28.3 ± 10.4
**BMI (kg/m^2)**	24.3 ± 2.41	24.5 ± 2.4
**Age at diagnosis (years)**	NA	15.3 ± 13.3
**Progression (years)**	NA	13.0 ± 7.0
**HbA1c (mmol/mol)**	NP	64.5 ± 5.0
**HbA1c (%)**	NP	8.1 ± 0.5

Data presented as mean±SD. No statistical differences were found between groups (Mann-Whitney test). F: female; M: male; BMI: Body Mass Index; NA: Not Applicable; HbA_{1c}: glycated hemoglobin; NP: Not Performed.

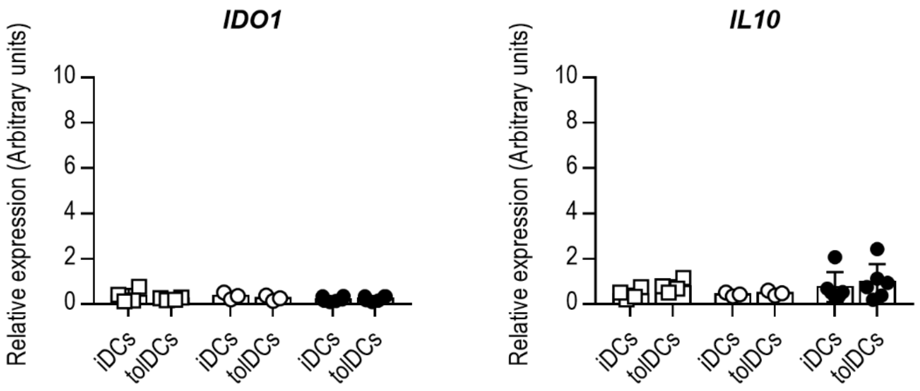

Figure S3. *IDO1* and *IL10* gene expression is detected at low levels in DCs. Expression of *IDO1* and *IL10* gene expression genes in immature DCs (iDCs) before and 4 hours after (tolDCs) coculture with PSAB-liposomes, analyzed by qPCR. White squares are control subjects (n = 4), white circles are patients at onset (n = 3-5), and black dots are patients with established disease (n = 6). Bars show the mean±SD. Gene expression signals were normalized to *GAPDH* expression. No significant differences were found when comparing culture conditions in the same group (Wilcoxon test), nor when comparing the same culture conditions in the three groups (Mann-Whitney test).
